# Editorial: Advances in integrative medicine for neurodegenerative diseases: from basic research to clinical practice

**DOI:** 10.3389/fneur.2023.1197641

**Published:** 2023-05-16

**Authors:** Shaonan Liu, Guoqing Zheng, Hansen Chen, Guowei Li, Xinfeng Guo

**Affiliations:** ^1^Guangdong Provincial Hospital of Chinese Medicine, Guangzhou University of Chinese Medicine, Guangzhou, China; ^2^Department of Neurology, The First Affiliated Hospital of Zhejiang Chinese Medical University (Zhejiang Provincial Hospital of Chinese Medicine), Hangzhou, China; ^3^Department of Neurosurgery, School of Medicine, Stanford University, Stanford, CA, United States; ^4^Guangdong Second Provincial General Hospital, Guangzhou, China

**Keywords:** integrative medicine, neurodegenerative disease, complementary and alternative medicine, preclinical and clinical study, Chinese medicine (CM)

Nesurodegenerative diseases (NDs) are disorders characterized by the progressive loss of neurons, which can result in motor dysfunctions and psychobehavioral manifestations such as ataxia and dementia (1). Alzheimer's disease (AD) as the most common NDs represents ~60%−70% of about 50 million people worldwide who suffer from dementia (2). The Research Topic aims to explore the therapeutic effect and mechanism action of integrative medicine of NDs for the improvement of patient healthcare, in order to stimulate further understanding and ultimately provide new methods for the prevention and treatment of NDs. For integrative medicine (IM), the topic mainly focuses on Chinese herbal medicine, acupuncture and related therapies, other Chinese medicine therapies based on modern clinical medicine. This collection spans different diseases such as AD and amyotrophic lateral sclerosis (ALS), and mainly focus on the mechanisms of neurodegeneration and treatment strategies.

In this volume, most studies focus on AD. Pathogenic mechanisms and new targeted drugs are still urgently needed although some progress has been made in this field. Zhang et al. performed an integrated analysis of the hub gene based on cuproptosis which is a copper-triggered modality of mitochondrial cell death by the bioinformatics approach for the diagnosis and treatment of AD. Seven hub genes including A4GALT, ALOX5AP, CLIC1, IFI30, LYZ, PLA1A, and PYGL were involved in phosphoribosyl pyrophosphate, lipid and glucose metabolism as revealed by GO analysis. Four of the seven cuproptosis genes (including IFI30, PLA1A, ALOX5AP, and A4GALT) can help with the clinical diagnosis of AD. These cuproptosis gene signatures may be an important diagnostic and prognostic indicator for AD. A similar bioinformatics analysis was conducted to explore the biomarkers and potential mechanisms that distinguish between Dementia with Lewy bodies (DLB) and Parkinson's disease dementia (PDD). Xu et al. identified seven upregulated genes, namely, SNAP25, GRIN2A, GABRG2, GABRA1, GRIA1, SLC17A6 and SYN1, which are involved in the heterogeneous pathogenesis of PDD and DLB. Blood pressure variability (BPV) has emerged as a novel risk factor for Alzheimer's disease, Yu et al. investigated the association of night BPV with brain atrophy and cognitive function changes from Korean Genome Epidemiology Study (KoGES). The results showed that high night systolic BPV was associated with temporal gray matter atrophy and impaired visual memory and verbal fluency. Subcortical vascular mild cognitive impairment (svMCI) is one of the most treatable cognitive impairments. Wang et al. explored the spontaneous brain activities regarding Chinese medicine deficiency patterns (DPs) and excess patterns (EPs) of svMCI patients based on fMRI data. The results found that the right middle frontal gyrus might serve as a brain response to endogenic cognitive impairments of DPs in svMCI patients. In addition to mechanisms exploration, several studies investigated the potential therapeutic strategies. Liu et al. found that the Chinese formula Liuwei Dihuang decoction could ameliorate cognitive dysfunction and hippocampal synaptic ultrastructure damage in aging mice by regulating lipid metabolism and oxidative stress via the microbiota-gut-brain axis. The current evidence showed that deficits of adult hippocampal neurogenesis (AHN) were the main hallmark of psychiatric diseases and neurodegeneration. Sun et al. conducted a review and found that exercise may be the ideal option to improve mitochondrial functions and AHN due to the relatively few safety concerns. Cao et al. summarized the studies about Alzheimer's drug development by the clinical trial registry platform. Sixteen compounds of disease-modifying therapies and symptomatic therapies such as gantenerumab, aducanumab, and others, may change the situation in China where there is no alternative drug for the treatment AD.

Two studies focus on ALS. Gong et al. explored the correlation between cerebrospinal fluid (CSF) and serum tau (t-tau, p-tau) in patients with ALS. Results suggest that CSF P-tau may be recognized as a potential cognition impairment biomarker in ALS. Liao et al. conducted a systematic review to explore the efficacy and safety of Chinese herbal medicine for the treatment of ALS. The findings suggest that the adjunct use of CHM can improve the ALS functional rating scale when compared with placebo or riluzole alone.

The collection of articles on this topic provides molecular mechanisms and clinical insights into neurodegenerative diseases, as well as the potential application of integrative medicine for neurodegenerative diseases.

## Author contributions

SL drafted the editorial. GZ, HC, GL, and XG reviewed the manuscript. All authors listed have made a substantial, direct, and intellectual contribution to the work and approved it for publication.
